# Platelet-Sourced TGF-β Promotes Th17 Responses and Enhances Airway Neutrophilia

**DOI:** 10.3390/biom15040482

**Published:** 2025-03-26

**Authors:** Ruoning Wang, Dandan Wu, Chunqing Wang, Amanda Livingston, Xiang Wu, Meilian Liu, Xuexian O. Yang

**Affiliations:** 1 Department of Molecular Genetics and Microbiology, University of New Mexico School of Medicine, Albuquerque, NM 87131, USA; ruwang@salud.unm.edu (R.W.); dwu@salud.unm.edu (D.W.); amanda.livingstonnnn@gmail.com (A.L.); wuxiang@csu.edu.cn (X.W.); 2 Department of Biochemistry and Molecular Biology, University of New Mexico School of Medicine, Albuquerque, NM 87131, USA; chuwang@salud.unm.edu (C.W.); meilianliu@salud.unm.edu (M.L.); 3 Department of Parasitology, School of Basic Medical Sciences, Xiangya School of Medicine, Central South University, Changsha 410013, China

**Keywords:** TH17, platelet, TGF-β, asthma, *Candida albicans*

## Abstract

Microbial, especially fungal, sensitization has been associated with the development and exacerbation of treatment-refractory neutrophilic asthma. Among the airway-inhabiting fungi, *Aspergillus fumigatus* and *Candida albicans* are the dominant species that elicit protective T helper (Th) 17 and other T cell responses, contributing to airway neutrophilia and steroid resistance. However, it is not fully understood how fungal airway colonization impacts the immunopathogenesis of asthma. Here, we used a neutrophilic asthma model induced by *C. albicans* to study the immune regulation of this disease. We found that intranasal administration of *C. albicans* induced platelet infiltration into the lung. Platelet-expressed latent TGF-β could be activated specifically by Th17 cells and drive the commitment, maintenance, and expansion of Th17 cells. In Candida-induced asthma, an adoptive transfer of platelets enhanced Th17 responses, increasing airway neutrophil influx. Thus, managing airway mycobiota and reducing platelet intrapulmonary infiltration may serve as a promising interventional approach.

## 1. Introduction

Asthma is a chronic inflammatory disorder manifested by airway hyperresponsiveness, airway hyperplasia, and elevated inflammation [1]. Over the past five decades, its prevalence and severity have increased globally. Allergic asthma, primarily characterized by Th2 and ILC2 dominance, accounts for approximately 60% of all cases of asthma. In this category, most type 2 (eosinophilic) asthma cases respond effectively to inhaled and oral corticosteroids, beta-agonists, and leukotriene modifiers. However, it is worth noting that a significant challenge remains in managing severe asthma cases that do not respond adequately to these treatments, demanding sophisticated mechanistic studies. Without known discriminators for steroid resistance, neutrophils and an imbalance of Th17/Treg cells have been related to steroid-resistant, severe, and exacerbation-prone asthma [2]. Interestingly, a subset of multi-drug resistance type 1 (MDR1)-expressing human Th17 cells is resistant to glucocorticoid treatment [3]. In addition, Th17 cytokine IL-17 upregulates glucocorticoid receptor-β (GR-β), a dominant-negative inhibitor of the functional GR-α that impacts glucocorticoid responsiveness [4,5].

Neutrophilic asthma has stronger associations with extrinsic factors such as air pollutants, infections, smoking, and obesity [6]. Enhanced exposure to aerosolized fungal spores and indoor molds has been linked to multiple chronic airway diseases, including the severity and hospitalization of asthma [7]. Compromised airways further contribute to the susceptibility of fungal sensitization. The breach in the physical integrity of the airway, debilitated immune system, and usage of antibiotics and glucocorticoids render individuals with asthma more vulnerable to recurrent and chronic airway mycosis [8]. Asthma exacerbated by fungal infection can be categorized into severe asthma with fungal sensitization (SAFS) and allergic bronchopulmonary mycosis (ABPM) based on disease severity. SAFS patients are airway colonized by fungi at lower concentrations than that seen in ABPM. ABPM can be further classified into allergic bronchopulmonary aspergillosis (ABPA), allergic bronchopulmonary candidiasis (ABPC), and others, based on the dominant fungal species involved [9]. Fungi are recognized as potent triggers of IgE-mediated sensitization and are capable of eliciting a Th17-driven immune response, both of which are believed to play a significant role in the pathogenesis of asthma [9,10]. Th cells serve as key conductors in directing adaptive immune responses against fungi, among which Th17 cells produce pro-inflammatory cytokines IL-17, IL-17F, and GM-CSF, driving airway neutrophilia [11,12]. Nevertheless, our understanding of fungi-induced Th cell responses and their contribution to protective immunity or chronic lung inflammation remains limited.

*C. albicans* airway colonization has been shown to induce protective Th17 and Th2 responses via recruitment and activation of platelets through candidalysin–GPIbα interaction [13]. Interestingly, Th17 cells mediate the antifungal immunity mainly through their cross-reactivity against Candida antigens [14]. Studies using multiple non-infectious and infectious disease models have proved the ability of platelets to actively migrate from blood into the lungs, liver, and spleen [15]. They express integrins and glycoprotein (GP) complexes to interact with the extracellular matrix and various myeloid cells, forming platelet–myeloid cell aggregates [15,16,17,18]. These aggregates are increased in the blood of asthmatics [18]. Platelet interaction modulates the recruitment and activities of neutrophils, eosinophils, and leukocytes. Platelet recruitment has been found to be important for restraining various fungal infections in the lungs and the pathogenesis of asthma, involving bronchoconstriction, airway hyperresponsiveness, and bronchial wall remodeling [19,20]. Interestingly, platelets activated by immune insults undergo autolysis rather than aggregation, highlighting a mechanism distinct from their role in the hemostatic response. Activated platelets secrete various mediators such as cytokines, chemokines, and immunomodulatory neurotransmitters, which orchestrate the activities of lymphocytes and leukocytes [13,19,21,22]. However, how fungi-initiated platelet infiltration participates in Th17 responses and airway neutrophilia remains limited.

TGF-β is the vital and pleiotropic cytokine regulating the balance between pro-inflammatory and anti-inflammatory T cells during adaptive immune response. It is generally considered to be immunoinhibitory, but it can also promote Th17 commitment in the context of a pro-inflammatory cytokine milieu [23]. Additionally, TGF-β contributes to airway fibrosis, oxidative stress, and the expression of GR-β [5,24,25,26]. TGF-β complexes with the latency-associated peptide (LAP) and maybe also the latent TGF-β binding protein (LTBP) [27]. This latent TGF-β requires protease, integrins, thrombospondin, and/or other factors to liberate its activity by removal of LAP, and active TGF-β modulates immune responses [28,29]. In this study, we found that Th17 cells released latent TGF-β from platelets that promoted the commitment, expansion, and maintenance of Th17 cells in vitro. Despite the fact that TGF-β can be produced by various types of cells, platelets have been considered as the primary source of TGF-β in both blood and organs [26]. Candida airway sensitization recruited free and platelet–myeloid aggregates into the lung. In a Candida-elicited airway inflammation model, adoptive transfer of platelets augmented Th17 responses and neutrophilic infiltration in the lungs independent of Candida product candidalysin. Thus, we uncovered a previously unacknowledged mechanism through which fungus-induced lung infiltration of platelets exacerbates neutrophilic airway reactions by providing TGF-β to amplify local Th17 responses. The results shed light on our understanding of the multifaceted role of platelets in the modulation of immune responses in various diseases, especially those driven by Th17 cells.

## 2. Materials and Methods

### 2.1. Mice

C57BL/6 (B6) mice were used in this study. All mice were housed in a specific pathogen-free animal facility (Animal Welfare Assurance # D16-00228). Sex- and age (6–12 weeks)-matched mice were used in all experiments. All animal experiments were conducted following the protocol (approval code: 22-201272) approved by the Institutional Animal Care and Use Committee of the University of New Mexico Health Sciences Center on 3 November 2022.

### 2.2. Induction of Candida-Mediated Airway Inflammation

In this study, 6–8-week-old mice were intranasally (i.n.) administered *C. albicans* extract (50 μg protein contents/mouse, prepared by sonicating heat-inactivated *C. albicans* in phosphate-buffered saline (PBS) and discarding the debris, 169246, Greer) on days 0 and 5. On day 7, the mice were euthanized by isoflurane overdose, and blood (100 μL/mouse) was collected by retro-orbital bleeding. The mice were then intracardiacally perfused with 2 mM EDTA until the lungs appeared visibly whitened. Bronchoalveolar lavage fluid (BALF), lungs, mediastinal lymph nodes, and spleens were immediately collected and analyzed for infiltrates and immune responses. Lungs were meshed in 1 mL of PBS using a 120-micron nylon mesh. After being pelleted and resuspended, the resulting cells were subjected to a 37% Percoll (17089101, GE Healthcare Life Sciences, Marlborough, MA, USA) density gradient with centrifugation at 10,000 rpm for 10 min to enrich myeloid cells in the lower layer and lymphocytes at the bottom for flow cytometry analysis. Both male and female mice were included in the experiments, but no significant gender differences were observed.

### 2.3. Adoptive Transfer of Platelets

To understand their role in airway TH17 responses, platelets were intratracheally (i.t.) transferred into the Candida-treated mice. In brief, mice were sensitized intranasally with 50 μL inactive *C. albicans* and 25 μg/mL ovalbumin (OVA) in PBS on days 0 and 2. Following sensitization, the mice were randomly assigned into two groups. On days 7 and 10, the mice in the control group received an intratracheal delivery of 50 μL of 25 μg/mL OVA in PBS, and the mice in the experiment group received an intratracheal transfer of 4 × 10^6^ platelets with 50 μL of 25 μg/mL OVA in PBS. On day 13, the mice were sacrificed, and the tissues were collected for analysis.

### 2.4. Isolation of Platelets

Blood up to 1 mL was collected from B6 mice by retro-orbital bleeding as a terminal procedure under anesthesia and anticoagulated with 40 μL of 500 mM EDTA. Platelets were isolated with 10,000 rpm, 10 min centrifugation with discontinuous gradients (1.068 g/mL and 1.072 g/mL) of platelet isolation medium Percoll. Briefly, the 1.123 g/mL stock isotonic Percoll (SIP) was prepared with 9 parts (*v*/*v*) of 1.130 g/mL Percoll (undiluted) mixed with 1 part of 1.058 g/mL 1.5 M NaCl, resulting in a density ($\rho_{SIP}$) of 1.1165 g/mL. Then, SIP was added to 1.0046 g/mL 0.15 M NaCl. The volumes required to obtain a solution of the desired density were calculated as follows:

$$V_{0.15M\;NaCl}=V_{SIP}\frac{\rho_{SIP}-\rho_{desired}}{\rho_{desired}-\rho_{0.15M\;NaCl}}$$

**Figure 1:**
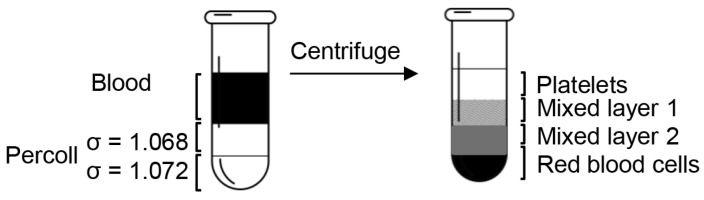
Diagram of the platelet isolation process. When the blood sample was centrifuged over the Percoll concentration gradients, it separated into four clearly defined layers: a platelet layer, two mixed layers, and a red blood cell layer.

After dilution, the final pH and density of Percoll were confirmed by a pH meter and a densitometer. The cushion of 200 μL Percoll of 1.068 g/mL was underlayered by penetrating the tip to the bottom of the 700 μL anticoagulated blood with slow injection, followed by 200 μL Percoll of 1.072 g/mL placed underneath the previous Percoll layer (Figure 1). After centrifugation at 10,000 rpm for 10 min, the blood was separated into four distinct layers.

### 2.5. Isolation of Splenic Dendritic Cells (DCs)

Splenocyte single-cell suspension was treated with ACK lysis buffer (0.1 mM EDTA, 150 mM NH_4_Cl, and 10 mM KHCO_3_) for two minutes at room temperature to remove red blood cells. The cells were subsequently incubated with anti-(α)-B220-biotin (RA3-6B2, BioLegend, San Diego, CA, USA), α-Thy-1.2-biotin (30-H12, eBioscience, Thermo Fisher Scientific, Waltham, MA, USA), and α-Ly6G (1A8-Ly6g, BioLegend) antibodies for 16 min with agitation to label B cells, T cells, and neutrophils. After washes, the cells were negatively selected using goat α-rat IgG magnetic beads (BM560, Bangs Laboratories, Fishers, IN, USA), enriching DCs. DC–platelet aggregates were then positively selected using biotinylated α-CD41α (MWReg30, eBioscience) and streptavidin magnetic beads (1420S, New England BioLabs, Ipswich, MA, USA); the remaining DCs were CD41α^−^ DCs (controls).

### 2.6. Th1, Th2, and Th17 Cell Differentiation and Co-Culture

Naïve CD4^+^CD25^−^CD62L^+^ T cells were selected using magnetic beads from the splenocytes and lymph node cells from B6 mice and differentiated in a Th1 (10 ng mL^−1^ IL-12 and 5 μg mL^−1^ α-IL-4), Th2 (5 ng mL^−1^ IL-4 and 5 μg mL^−1^ α-IFN-γ), or Th17 (2 ng mL^−1^ TGF-β, 10 ng mL^−1^ IL-6, 2 μg mL^−1^ α-IFN-γ, and 2 μg mL^−1^ α-IL4)-polarizing condition on an α-CD3 and α-CD28 (1 μg mL^−1^, each) coated plate for 4 days. In some experiments, purified platelets at indicated amounts and/or 10 μg mL^−1^ α-TGF-β (1D11.16.8, Bioxcell, Lebanon, NH, USA) were added. When splenic DCs were used to differentiate Th17 cells, the culture was supplied with 10 ng mL^−1^ IL-6, 2 μg mL^−1^ α-IFN-γ, and 2 μg mL^−1^ α-IL4 without exogenous TGF-β in the presence of plate-bound α-CD3. For Th17 cell maintenance and expansion, the cells were cultured with platelets, TGF-β, or medium in the presence of IL-6 on an α-CD3 and α-CD28 coated plate for 3 days.

To determine whether Th cells can activate platelet surface-bound latent TGF-β, differentiated Th cells were co-cultured with platelets. Prior to co-culture, Th cells were activated by incubating them on an α-CD3-coated plate for two hours. Concurrently, platelets and DCs were isolated, counted, and maintained on ice to preserve their viability. Once all cell preparations were complete, the cells were combined at the appropriate cell densities and co-cultured in a 24-well plate. A subset of cells without culture was fixed and used as control groups for subsequent analysis. The co-culture was maintained in a humidified incubator at 37 °C with 5% CO_2_ for the specified durations.

### 2.7. Flow Cytometry

Antibodies against CD45.2 (104), CD11b (M1/70), CD11c (N418), Ly6G (1A8-Ly6g), CD41α (MWReg30), CD4 (GK1.5), IL-4 (11B11), IL-17A (eBio17B7), and FoxP3 (FJK-16s) were purchased from eBioscience, and CD4 (RM4-5), IFN-γ (XMG1.2), and LAP (TW7-16B4) were from BioLegend. For intracellular staining, cells were first stained with α-CD4 antibody and then fixed with 2% paraformaldehyde for 10 min at room temperature. After fixation and wash, the cells were permeabilized and stained with the appropriate antibodies in a permeabilization buffer containing 0.1% Saponin (S7900, Sigma-Aldrich, St. Louis, MO, USA) and 0.1% BSA in PBS. The staining was conducted at 4 °C, with 1–2 gentle agitations, for 50 min when targeting cytosolic cytokines (such as IFN-γ and IL-17) or for 70 min when targeting the transcription factor Foxp3. Finally, the stained cells were analyzed using an Attune NxT Flow Cytometer (Thermo Fisher Scientific, Waltham, MA, USA). Data were processed by FlowJo software (version 10.8.1, FlowJo, LLC). For imaging flow cytometry, cells were labeled with antibodies against CD11b, CD11c, Ly6G, and CD41α. Single-cell images were acquired with Amnis^®^ ImageStream^®^ X MkII (Luminex Corporation, Austin, TX, USA), and collected data were analyzed with IDEAS 5.0 software (Luminex).

### 2.8. Statistical Analysis

Data were analyzed using Microsoft Excel (version 365, Microsoft Corporation, Redmond, WA, USA), GraphPad Prism (version 8.4.2, GraphPad Software, San Diego, CA, USA), or R. Descriptive statistics, including means and standard deviations, were calculated for all variables. Student’s *t*-tests (for 2 groups) or one-way analysis of variance (ANOVA) with post hoc Tukey’s honest significant difference (HSD) test (for multiple groups) were employed as appropriate for comparisons between groups based on the data distribution and sample size with a significance level set at *p* ≤ 0.05.

## 3. Results

### 3.1. Platelets and Platelet–Myeloid Aggregates Infiltrate Candida-Challenged Airways

**Figure 2:**
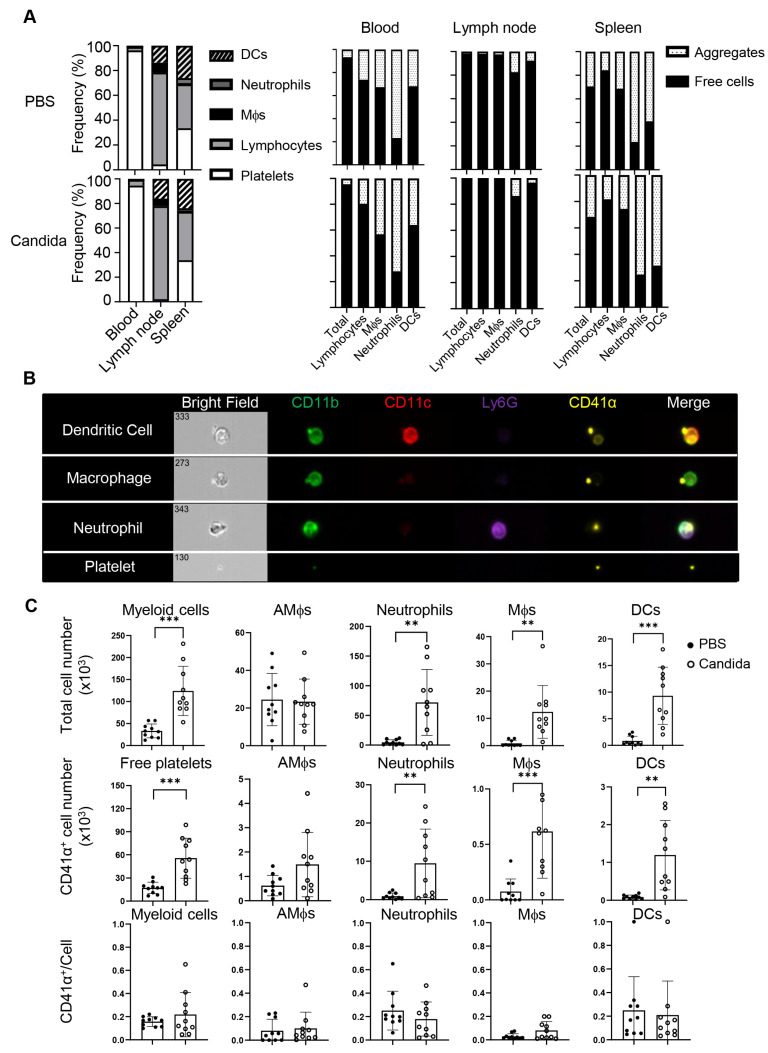
Tissue distribution of myeloid aggregates and free platelets following *C. albicans* challenges. Mice were intranasally (i.n.) administered with PBS or inactivated *C. albicans* on day 0 and day 5. Blood, lymph nodes, spleens, and lungs were collected on day 7. (**A**) Left, frequencies of DCs, neutrophils, macrophages, lymphocytes, and platelets in blood, lymph nodes, and spleens of control or Candida-sensitized mice. Right, percentages of free platelets, platelet–myeloid aggregates, and myeloid cells. (**B**) Representative images of platelet–myeloid aggregates in *C. albicans*-challenged mice. (**C**) Candida sensitization promotes lung infiltration of platelets, myeloid cells, and platelet–myeloid aggregates. Mϕs, macrophages; AMϕs, alveolar macrophages; DCs, dendritic cells. N = 10 per group. The data shown are mean ± SD. Student’s *t*-test, ** *p* < 0.01, *** *p* < 0.001.

Platelets have been shown to actively migrate into many tissues, including the lung [15,16,19], and thus may influence local inflammatory responses. To better understand how asthmatic reactions influence the distribution of platelets, we intranasally challenged C57BL/6 (B6) mice twice with inactivated *C. albicans* or vehicle and profiled platelets and platelet-containing aggregates in the blood, spleens, mediastinal lymph nodes (mdLNs), and lungs of these mice. The Candida challenge increased the ratio of lymphocytes in the spleens, associated with splenomegaly, but not in the blood and mdLNs (Figure 2A, left). There were no significant differences in platelet distribution in lymph nodes or spleen between vehicle- and Candida-treated mice (Figure 2A, right). While blood was dominated by free platelets, over 80% of myeloid cells and 20% or fewer lymphocytes in the blood were decorated with platelets. Although platelet–leukocyte aggregates are increased in the blood of asthmatics [17], we did not observe a similar alteration in the blood of the above mice with an acute *C. albicans*-challenged program. Interestingly, lymphocytes predominated in lymph nodes, where most lymphocytes and myeloid cells were devoid of platelets. Unlike mdLNs, the spleen exhibited a rich presence of both lymphocytes and myeloid cells. Notably, the ratio of platelet aggregates to free cells in the spleen mirrored the blood composition. Thus, the distribution of conjugates and free platelets in the spleen but not the lymph nodes reflects the abundance of blood therein.

The scenario differed within the lungs. Imaging flow cytometry revealed that *C. albicans* challenge induced migration of free and conjugated platelets into the lungs, which were perfused with 2 mM EDTA to minimize contamination of platelets from the bloodstream (Figure 2B), which was hard to observe in the control lungs. *C. albicans*-challenged mice exhibited a significant increase in myeloid cells (CD45.2^+^) compared to the control group, including alveolar macrophages (AM, CD11b^lo^ CD11c^hi^), neutrophils (CD11b^+^ Ly6G^+^), macrophages (CD11b^+^ CD11c^lo^), and dendritic cells (DCs, CD11b^+^ CD11c^+^) (Figure 2B,C). In addition to free platelet infiltration, approximately 20% of myeloid cells (mainly neutrophils and macrophages) were associated with platelets. Given the antigen-presenting properties of DCs and macrophages, the platelet–myeloid cell aggregates may serve as a platform to modulate T cell responses. In summary, Candida challenge induces massive airway infiltration of platelets in the form of free platelets and platelet–myeloid aggregates.

### 3.2. Th17 Cells Activate Latent Surface TGF-β on Platelets

**Figure 3:**
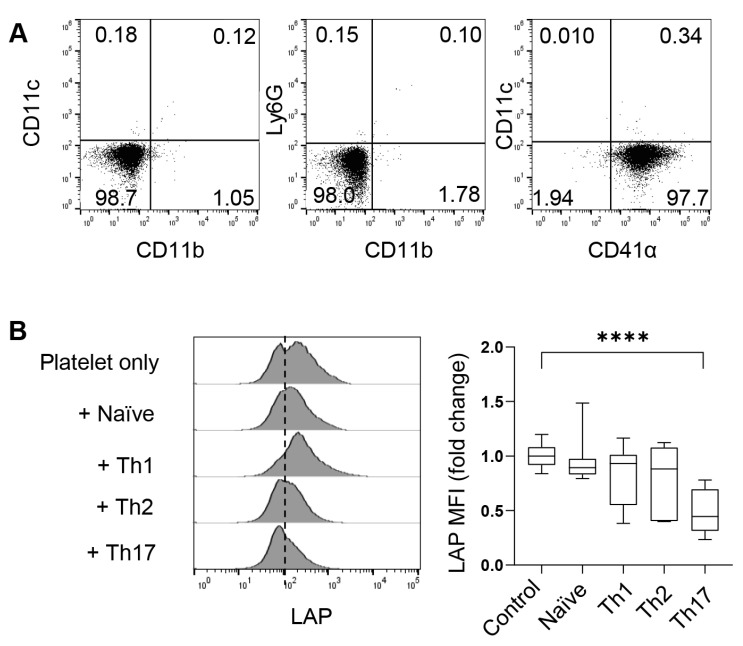
CD4^+^ T cells activate platelet surface-bound TGF-β. (**A**) Flow cytometric characterization of platelets in the upper layer of Figure 1. (**B**) Flow cytometry of LAP on platelets co-cultured with CD4^+^ naïve T cells or indicated T helper cells for four hours. N = 11–22 per group. The data shown are mean ± SD. One-way ANOVA with post hoc Tukey HSD test, **** *p* < 0.0001.

To investigate the role of platelets through in vitro experimental approaches, we isolated free platelets from the blood using Percoll gradients. After centrifugation, the blood formed four layers (Figure 1). CD41α (also known as integrin αIIb or GPIIb) is a common surface marker of platelets and megakaryocytes. As revealed by flow cytometry, free CD41α^+^ platelets dominated the cell population in the top layer, comprising less than <2% of neutrophils, macrophages, or dendritic cells (DCs) (Figure 3A). The second layer from the top, appearing white and blurry, contained a mixture of white blood cells, including some free platelets, and the third layer was red and transparent with a mixture of blood cells and a few free platelets. The pellet of red blood cells formed the bottom layer.

As shown in Figure 2, platelets can migrate into the lung and even lymphoid organs, such as the spleen and mdLNs. In these organs, platelets present TGF-β (most commonly TGF-β1) on the cell surface or secrete it in alpha granules. To understand whether Th cells can activate latent TGF-β on the surface of platelets, we co-cultured platelets with naive CD4^+^ T cells or various in vitro-differentiated Th cells for four hours and examined the cleavage (loss) of LAP on platelets. We found that co-culture with Th17 cells significantly reduced the mean fluorescence intensity (MFI) of LAP on the surface of platelets. Th17 were potent activators of LAP compared to Th1, Th2, and naïve T cells (Figure 3B). Therefore, Th17 cells are capable of cleaving surface LAP and thus releasing TGF-β, which may in turn influence the differentiation and functionality of Th17 cells.

### 3.3. Platelets Promote Th17 Polarization, Maintenance, and Expansion

**Figure 4:**
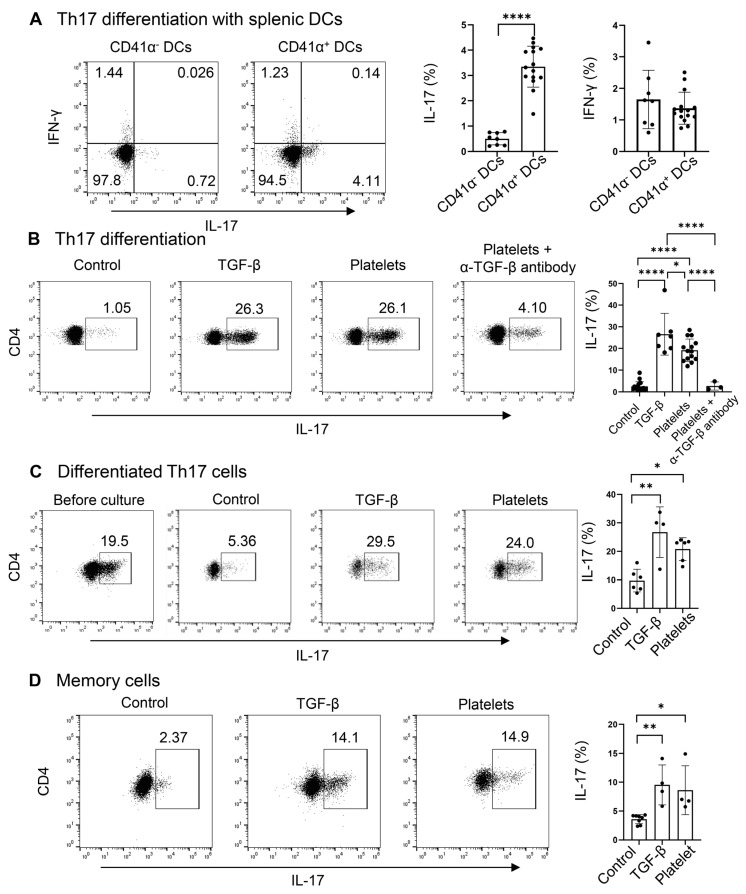
Platelets promote the differentiation, maintenance, and expansion of Th17 cells. (**A**) Flow cytometry of Th17 cells differentiated with DCs. Naive CD4^+^ T cells were cultured with purified splenic CD41α^+^ (platelet–DC aggregates) or CD41α^−^ DCs on an α-CD3-coated plate in the presence of IL-6, α-IFN-γ, and α-IL4. DCs, dendritic cells (**B**) Flow cytometry of Th17 cells differentiated in a DC-free system. Naive CD4^+^ T cells were cultured with purified platelets, recombinant TGF-β, or medium on an α-CD3 and α-CD28 coated plate in the presence of IL-6, α-IFN-γ, and α-IL4 with or without α-TGF-β. (**C**) Flow cytometry of differentiated Th17 cells after culture with platelets, recombinant TGF-β, or medium on an α-CD3 and α-CD28 coated plate in the presence of IL-6. (**D**) Flow cytometry of memory Th17 cells. Splenic CD4^+^CD62L^−^CD44^+^ were cultured with platelet, recombinant TGF-β, or medium on an α-CD3- and α-CD28-coated plate in the presence of IL-6. N = 4–15 per group. The data shown are mean ± SD. Student’s *t*-test (**A**) and one-way ANOVA with post hoc Tukey HSD Test (**B**–**D**), * *p* < 0.05, ** *p* < 0.01, **** *p* < 0.0001.

Due to the presence of a high percentage and large number of platelet-adherent DCs in the spleens, regardless of the treatment with Candida or PBS (Figure 2A), we hypothesized that platelets may drive the differentiation of naïve CD4^+^ T cells into Th17 cells. Since CD4^+^ T cells, especially Th17 cells, activate the latent form of TGF-β on the surface of platelets (Figure 3), we examined whether DC-bound platelets might provide TGF-β for Th17 cell differentiation. We investigated the role of CD41 α^+^ splenic DCs in driving Th17 commitment in CD4^+^ naïve T cells, using TGF-β as a positive control. CD41α^+^ DCs significantly enhanced IL-17^+^ Th17 (but not IFN-γ^+^ Th1) cell polarization of naïve CD4^+^ T cells compared to CD41α^−^ DCs (Figure 4A), suggesting that the DC-conjugated platelets may participate in Th17 cell polarization by providing TGF-β.

We have shown above that the latent TGF-β on platelets can be activated by Th17 cells themselves; therefore, we asked if platelets can substitute TGF-β in priming Th17 cells. To explore this, we replaced TGF-β with platelets during Th17 cell differentiation in a DC-free system. Platelets and T cells were co-cultured at a 1:1 ratio. We observed that in the presence of IL-6 plus α-IFN-γ and α-IL-4, there were a few percent of IL-17^+^ cells, whereas the addition of TGF-β greatly enhanced the frequencies of IL-17^+^ cells (Figure 4B). Surprisingly, platelets stimulated Th17 differentiation akin to the effect of TGF-β, which can be negated by the neutralizing antibody against TGF-β (Figure 4B). Under the Th1 condition, both TGF-β and platelets suppress Th1 cell differentiation, in agreement with the previous study [23]. In addition, we co-cultured in vitro-differentiated Th17 cells (about 20% of IL-17^+^ cells) with IL-6 on an α-CD3 and α-CD28-coated plate for 3 days. We found that a part of the cells lost the expression of IL-17 in the IL-6 alone (control) condition. Interestingly, the supplement of platelets at a 1:1 ratio, similar to that of TGF-β, could maintain the cytokine secretion from Th17 cells (Figure 4C). When splenic memory CD4^+^ T cells were co-cultured with TGF-β or platelets or a vehicle in the presence of IL-6, we observed that like TGF-β, platelets could expand IL-17^+^ cells compared to the control culture (Figure 4D). In summary, platelets, as a source of TGF-β, sufficiently supported Th17 differentiation, maintenance, and expansion.

### 3.4. Platelets Regulate the Th17-Treg Balance

**Figure 5:**
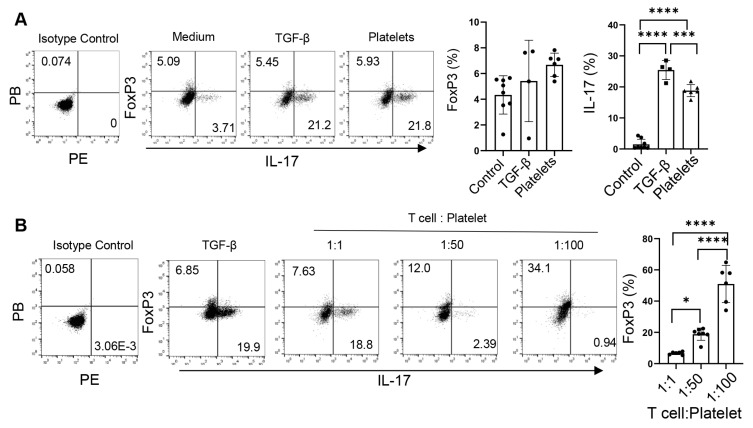
Platelets regulate the balance of Th17 and Treg cells. (**A**) Foxp3 expression of CD4^+^ T cells differentiated with purified platelets, recombinant TGF-β, or medium on an α-CD3 and α-CD28 coated plate in the presence of IL-6, α-IFN-γ, and α-IL4. (**B**) Flow cytometry of naïve CD4^+^ T cells differentiated with various amounts of purified platelets on an α-CD3 and α-CD28 coated plate in the presence of IL-6, α-IFN-γ, and α-IL4; 1:100: 1 naïve CD4^+^ T cells: 100 platelets. N = 4–8 per group. The data shown are mean ± SD. One-way ANOVA with post hoc Tukey HSD test, * *p* < 0.05, *** *p* < 0.001, **** *p* < 0.0001.

The fact that TGF-β not only regulates the commitment of Th17 cells but also controls the balance of Treg vs. Th17 cell development [30,31] prompts us to examine the role of platelets as a source of TGF-β in the reciprocal development of these two lineages. Under the Th17 conditions, TGF-β slightly increased the frequencies of both IL-17^+^ and Foxp3^+^ (Treg) cells, whereas equivalent numbers of platelets bolstered the percentages of IL-17^+^ cells but did not alter those of Foxp3^+^ cells (Figure 5A). Interestingly, increases in the abundances of platelets led to decreases in the frequencies of IL-17^+^ cells but elevations in Foxp3^+^ cells (Figure 5B), suggesting that platelets supplement TGF-β in modulating the Th17-Treg balance [30,31].

### 3.5. Platelets Augment Th17 Responses in Candida Airway Inflammation

**Figure 6:**
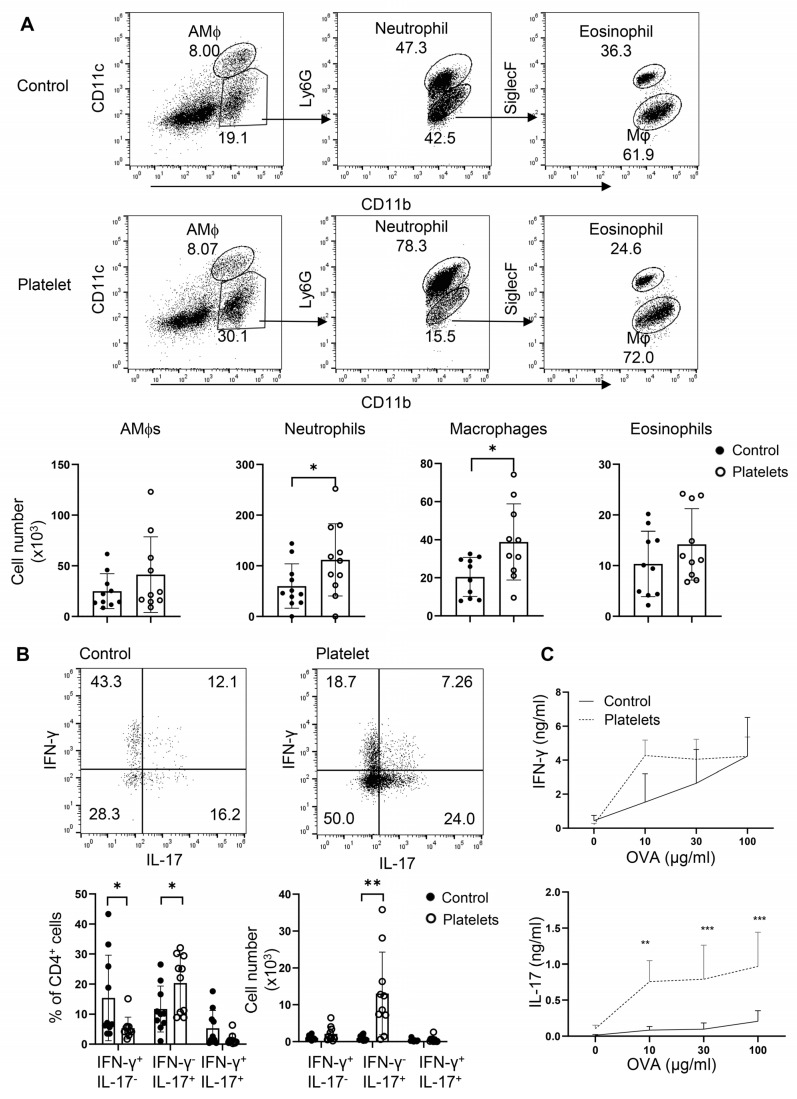
Adoptive transfer of platelets intensifies Th17 responses and promotes airway neutrophilia. Mice were sensitized i.n. with inactive *C. albicans* and OVA. Platelets or PBS (control) were then i.t. transferred to the resulting mice. (**A**) Flow cytometry of myeloid cells in BALF. Bottom, statistical analysis of numbers of alveolar macrophages (AMϕs), neutrophils, macrophages, and eosinophils. (**B**) Flow cytometry of lung infiltrating Th1 and Th17 cells. (**C**) ELISA of IFN-γ and IL-17 in OVA-recall supernatant of mediastinal lymph node cells. N = 9–10 per group. The data shown are mean + SD. Student’s *t*-test, * *p* < 0.05, ** *p* < 0.01, *** *p* < 0.001.

Platelets have been shown to play a protective role in airway anti-*C. albicans* immunity, in which candidalysin, a peptide toxin produced by Candida, targets GP1bα on platelets to secrete the Wnt antagonist Dickkopf-1 that promotes Th2 and Th17 responses [13]. However, it is unclear whether, in the absence of candidalysin (or *C. albicans*), platelets can support Th cell responses in vivo. To address this, we employed an adoptive transfer of platelets or a vehicle in a neutrophilic fungal asthma model. In brief, B6 mice were challenged with *C. albicans* extract and OVA, randomly split into two groups, and i.t. administered either purified platelets combined with OVA or OVA alone. Transfer of platelets significantly elevated the cell counts of neutrophils and macrophages in the BALF (Figure 6A). As expected, administration of platelets led to an increase in the frequencies and numbers of IL-17^+^IFN-γ^−^ Th17 cells with a trend of decrease in the percentages but not numbers of IFN-γ^+^ Th1 cells and IL-17^+^IFN-γ^+^ Th1/Th17 hybrid cells (Figure 6B). Conversely, a supplement of platelets bolstered OVA-specific Th17 but not Th1 responses as revealed by ELISA in OVA-recalled culture supernatant of mdLN cells (Figure 6C). Thus, platelets promote Th17 responses and airway neutrophilia independent of candidalysin from *C. albicans*. In summary, platelets may play an essential role in neutrophilic fungal asthma via shedding their surface-bound TGF-β to participate in Th17 responses.

## 4. Discussion

Despite the role of Th17-type responses in the etiology of neutrophilic severe asthma, the detailed regulation of these responses remains poorly understood. Enhanced exposure to aeroallergens, such as house dust mites, fungi, bacteria, and pollen, has been linked to asthma severity and hospitalization, especially in those with damaged airways [7,32,33,34]. *Schizophyllum commune*, *A. versicolor*, *A. fumigatus, C. albicans*, and *Clostridium* have been reported to induce neutrophilic asthma in the laboratory [6,35,36]. Fungal sensitization is frequently observed in patients with underlying lung conditions, especially among those with severe asthma, cystic fibrosis, and bronchiectasis, involving a sophisticated interaction between the immune system, fungal sensitivity, and asthma severity [8,10]. Nonetheless, research on fungal community has lagged behind studies on bacteria, with the lung being overlooked relative to the gut and other organs [9]. This may be partially due to the relatively rare prevalence of fungal infection, except for the opportunistic infection seen in immune-compromised subjects, compared to bacterial infection.

Our findings demonstrate that Candida challenge triggers a significant influx of platelets into the airways, comprising both free platelets and platelet–myeloid aggregates. Given the abundance of latent membrane-bound TGF-β on platelets, this robust platelet infiltration potentially influences the pathophysiology of neutrophilic fungal asthma. In Th2-high asthma, TGF-β is derived from multiple sources, but the overall immune response remains Th2-dominant, which may be due to a lack of strong Th17 skewing conditions. Numerous studies have associated Th2-low asthma with infections, obesity, air pollution, smoking, and other adverse environmental factors. Among these, we are particularly interested in how fungal sensitization alters the lung immune microenvironment, specifically through the recruitment of TGF-β-bearing platelets. Platelets, especially platelet–myeloid aggregates that contain antigen-presenting DCs and macrophages, are accessible to Th17 cells and may participate in promoting a Th17-dominant immune response. In this context, platelet-sourced TGF-β, after release, serves as a key mediator that amplifies local Th17 responses in the lung. The heightened Th17 responses drive neutrophilic inflammation, revealing a link between the infiltration of platelets and the development or exacerbation of fungal asthma. Together, these insights underscore the importance of platelets as potential regulators of immune pathways in this disease, offering new avenues for therapeutic exploration.

The IL-17/IL-17R signaling pathway is essential in host defense against extracellular pathogens, including *C. albicans* [37,38]. Among airway-inhabiting fungi, *C. albicans* is well recognized for its ability to elicit a Th17 response, which is, at least in part, through induction of the Th17-inducing cytokine IL-23 [39,40], playing a dual role in clearing mucosal *C. albicans* infections and contributing to treatment-refractory asthma [40]. In this context, gut dysbiosis of Candida also promotes asthmatic reactions [14,41]. Recently, *C. albicans* airway colonization has been shown to elicit protective Th17 and Th2 responses via recruitment and activation of platelets [13]. The interaction between Candida’s candidalysin with GPIbα induces the release of Dkk-1 from platelets, which antagonizes Wnt signaling and favors Th17 and Th2 cell responses. Several clinical trials have demonstrated that antimycotics significantly improved the quality of life for SAFS patients and reduced IgE concentrations [42,43]. A proposed mechanism for this improvement is that antimycotics decrease the fungal burden in the airways. This reduction in fungal load leads to fewer infiltrated platelets, thereby diminishing the resource of TGF-β in the lung. Consequently, this results in a downregulation of Th17 (and Th2) responses, contributing to the alleviation of symptoms and improved patient outcomes.

Platelets survey blood vessels and repair their damage. Moreover, activated platelets produce an array of mediators such as cytokines, chemokines, lipids, and neurotransmitters, modulating immune function [13,19,21,22]. Among the platelet cytokines, TGF-β modulates adaptive immune responses by inhibiting Th1 and Th2 and promoting Treg cell commitment. In addition, TGF-β induces Th17 cells in the presence of pro-inflammatory cytokines, such as IL-6 and IL-23, but restricts Treg cells [23,30,31]. Platelets bear a latent form of TGF-β on their cell surface that must be released before it can act on its target cells. Treg-sourced TGF-β can be activated by T cell-expressed integrin αvβ8 and participates in Th17 and inducible Treg differentiation [28,44]. This process likely requires a metalloproteinase (MMP) activity, as the pan MMP inhibitor blocks the effect of the integrin αvβ8 [45,46]. A recent study showed that ADAM9 is specifically expressed by Th17 cells and promotes Th17 differentiation through cleaving latent TGF-β [47]. Thus, T cell-expressed integrins and proteases are, at least in part, responsible for activating latent TGF-β and driving Th17 responses [48]. In line, we found that activated (especially Th17) but not naïve CD4^+^ T cells could activate latent TGF-β on the surface of platelets; platelets substituted recombinant TGF-β in the induction and maintenance of Th17 cells in vitro in a dose-dependent manner. Therefore, platelets, one of the major TGF-β reservoirs, directly participate in Th17 responses.

The initial commitment of Th cells occurs in lymphoid tissue, such as the draining lymph nodes and spleen, where antigen-presenting cells, mainly DCs, activate and instruct naïve CD4^+^ T cells towards effector Th cells. Th17 development is antagonized by the products of the Th1 and Th2 lineages [49,50]. TGF-β inhibits both Th1 and Th2 activities and, in the presence of a pro-inflammatory cytokine milieu (such as IL-6, IL-23, TNFα, and IL-1β), promotes Th17 commitment but diminishes Treg cell development [23]. Several common aeroallergens have been reported to induce IL-6 production in airway epithelial cells [51]. We observed a few platelet-DC aggregates in lymph nodes, but many of these were in the spleen; in vitro, platelet-bearing DCs were better inducers of Th17 cells than platelet-free DCs, suggesting that platelets may be involved in Th17 cell commitment in vivo. Using an adoptive transfer approach, we found that i.t. administration of platelets bolstered pulmonary Th17 responses, leading to enhanced airway neutrophilic influx. However, we cannot rule out the involvement of DC-sourced TGF-β in this model. *C. albicans* has been shown to induce Th17 cells via activating the Dectin-1-Card9 pathway in bone marrow-derived DCs [52,53], which depends on TGF-β, as neutralizing TGF-β diminishes the effects of *C. albicans* on the induction of Th17 cells [53]. In addition, elevated secretion of TGF-β by alveolar macrophages, airway eosinophils, and fibroblasts is observed in asthma patients, which may also participate in pulmonary Th17 responses [24,54,55]. Even so, *C. albicans* airway sensitization recruits platelets into the lung; as a consequence, we observed that platelets may serve as an important source of TGF-β for local Th17 cell maintenance and expansion.

## 5. Conclusions

Our findings bridge a crucial gap in understanding the role of platelets in the host–pathogen interaction-elicited adaptive immunity and pathophysiology of severe fungal asthma. By delving into the mechanisms involving platelet-sourced TGF-β and Th17 maintenance and expansion, this research delineates the inner connections between neutrophilic asthma and airway fungi colonization, aiding in the design of therapeutic options.

## Data Availability

All data generated or analyzed during this study are included in this article.
